# Illicit use of methadone and buprenorphine among adolescents and young adults in Sweden

**DOI:** 10.1186/1477-7517-10-27

**Published:** 2013-10-18

**Authors:** Torkel Richert, Björn Johnson

**Affiliations:** 1 Department of Social Work, Malmö University, Malmö, Sweden

**Keywords:** Methadone, Buprenorphine, Illicit use, Adolescents, Young adults, Drug career, Diversion

## Abstract

**Background:**

Illicit use of methadone and buprenorphine has been described as a growing problem in Sweden in recent years, and has been associated with an increased drug-related mortality. Critics claim that the substances have become popular among adolescents and that they function as a gateway to heroin use. The aim of this study is to investigate, firstly, the extent to which illicit use of methadone and buprenorphine occurs among adolescents and young adults in Sweden, and secondly, at what stage in a user’s drug career these substances tend to appear.

**Methods:**

The study is based on surveys and structured interviews on drug use among various populations of young people, in addition to qualitative interviews with 86 informants who, in their professional capacity, encounter adolescents or young adults who are using illicit drugs.

**Results:**

Illicit use of methadone and buprenorphine is rare among young people in Sweden. According to high school surveys, less than 0.1% have tried these substances. Among young drug users in general, few have tried the substances, and there is nothing to indicate that they act as gateway drugs. Among adolescents and young adults with severe drug problems, however, the illicit use of methadone and buprenorphine is more common (54% in a compulsory care sample). These substances normally enter the drug career late, and few use them as their main drug of choice. Other prescription drugs, like benzodiazepines and tramadol, are used by adolescents to a far greater extent. Diversion and illicit use of methadone and buprenorphine is not seen as a serious problem by the professionals interviewed. A general view is that the substances are mainly used by people with a heroin or polydrug addiction, often for “self-medication” purposes. However, several informants express concern that methadone and buprenorphine may cause fatalities among young drug users without an opioid tolerance.

**Conclusions:**

Illicit use of methadone and buprenorphine among young drug users is not a widespread problem in Sweden. Harm-reduction measures should target drug users with more severe problems, among whom illicit use of methadone and buprenorphine is more common and pose a medical risk. Illicit use of other prescription drugs, which are less controlled and more widely used by young people, is an important issue for further research.

## Introduction

Methadone and buprenorphine are secure and effective pharmaceutical drugs when used for opioid substitution treatment (OST) [1-3]. However, if the substances are used in a non-medical fashion, they can cause serious problems. Methadone is highly toxic, and may be lethal for anyone who has not developed a sufficient opiate/opioid tolerance [4,5]. Buprenorphine is less potent, but if mixed with alcohol or sedatives there is still a great risk of polydrug intoxication [6,7]. Both substances are highly addictive, and attractive on the illicit drug market [8,9].

In recent years, as OST has expanded in Sweden, attention has been drawn to illicit use of methadone and buprenorphine as a growing problem. ^a^The number of deaths related to methadone and buprenorphine have increased substantially, and in 2010 they exceeded heroin-related deaths for the first time [10]. Diversion from treatment programs has been suggested as a plausible explanation [11]. Moreover, critical voices in the debate have contended that the substances have become popular among and widely used by adolescents, that there is a risk of young people with no history of drug use becoming addicted to the substances, and that they provide gateway drugs to heroin use [12].

Among other things, the debate has led to a questioning of the legitimacy of OST, stricter controls in some treatment programs, and the withdrawal of the buprenorphine-based prescription drug Subutex from the market. This decision was taken without any existing research into the phenomenon. No research has been conducted regarding the extent to which methadone and buprenorphine are being diverted from the Swedish OST programs, the structure of the illicit market for these substances, or to what extent the substances come into the hands of young people without a drug addiction. A couple of Swedish studies of the illicit use of buprenorphine have been conducted [13,14]. Both focus exclusively on injecting drug users (IDUs), and indicate that, within this community, such use is very common. The most common reason for using the substance outside the treatment system was to alleviate heroin withdrawal symptoms, in addition to performing self-detoxification or managing opioid substitution on your own.

Whether diversion and illicit use of methadone and buprenorphine should be seen as a serious problem or not, depends not only on the extent of these phenomena, but also on the issue of which populations are using the substances and for what purpose. If these substances are mainly used by opioid IDUs for self-medication purposes, this may be seen as a less serious problem than if the substances to a great extent are used by adolescents and young adults early on in their drug careers.

The aim of this study is to investigate, firstly, the extent to which illicit use of methadone and buprenorphine occurs among adolescents and young adults in Sweden, and secondly, at what stage in a user’s drug career these substances tend to appear. In addition, we also investigate the extent to which diversion and illicit use of substances from the treatment programs is perceived as a serious problem by professionals who, in their line of work, encounter young people who use drugs.

There is a growing body of international research into illicit use of methadone and buprenorphine. However, this research has mainly focused on IDUs, or users admitted to OST programs. The first studies into illicit use of methadone were conducted in the US in the mid 1970s, some years after the rapid expansion of methadone clinics throughout the country. Ethnographic field studies from the middle of that decade paint a picture of how methadone had been integrated into the drug user environment and the street culture, and how it had become a prominent street drug [15,16]. More recent studies indicate that illicit methadone use is very common among opiate users—mainly among older drug users, users who have previously undergone OST, and people who recently have completed detoxification [17]. The lifetime prevalence of illicit methadone use among opiate users outside treatment has varied between 17% and 73% in different studies [17-20], but a high prevalence is the rule rather than the exception.

Diversion and illicit use of buprenorphine is the subject of a recently published research review [9]. The review shows that buprenorphine, like methadone, is a commonly used substance among IDUs and people with a heroin addiction in a great number of countries all over the world. Two countries, Finland and France, stand out in this context. A study from Finland shows that buprenorphine is the most commonly used opiate/opioid, and that 97% of the people applying for OST have buprenorphine as their main drug [21]. Studies from France indicate that a majority of IDUs either inject only buprenorphine, or are using the drug as part of a polydrug addiction, primarily in conjunction with heroin or cocaine [22,23].

Internationally there is also a dearth of knowledge as regards the extent to which methadone and buprenorphine are used by adolescents or young adults. An Irish study which investigated the journey into injecting drug use among a cohort of 104 young adults in OST showed that only 3% were introduced to opioid dependence via methadone (96% were introduced via heroin) [24]. A couple of studies indicate that, for some individuals, buprenorphine may play a major role in their drug career. In a survey study of intravenous drug users in the country of Georgia [25], 11.5% claimed that buprenorphine was the first drug to which they developed an addiction. An Indian study show that when transitioning to intravenous drug use, users tend to prefer buprenorphine instead of heroin [26]. However, these studies too are based on adults with severe drug problems.

## Methods

In order to investigate the extent of the illicit use of methadone and buprenorphine among various groups of adolescents and young adults in Sweden, we have collected data covering three populations: (1) a general population of high school students, (2) adolescents who use illegal drugs, and (3) adolescents and young adults with severe drug problems (many of them would classify at the severe end of the substance use disorder spectrum). By including populations with different experiences of drug use, we hope to get a picture of how common these substances are in comparison to other drugs, and at what stage in a user’s drug career they tend to be introduced.

We have compiled qualitative as well as quantitative data material about the three populations. By using different types of data—data triangulation—we are hoping to provide a comprehensive view of the phenomenon. The populations and the data sets are summarized in Table 1.

**Table 1:** Summary of populations and data sets

	**Quantitative data**	**Qualitative data**
General population of high school students	Survey data on drug use among Swedish high school students (CAN)	Interviews with high school counselors
Young drug users	Structured interview data from outpatient drug treatment units (UngDOK)	Interviews with police officers (adolescent units and drug squads), outreach/social street workers, youth workers, social workers within the police force, outpatient drug treatment staff, NGO-representatives
Adolescents and young adults with severe drug problems	Structured interview data from compulsory care units (SiS)	Interviews with addiction treatment staff (detox units, institutional care, compulsory care) and police officers (drug squads)

### Quantitative data

Our quantitative data is gathered from three data sets which we have been given access to via the organizations and authorities that assemble them. As substances, methadone and buprenorphine are only infrequently separately specified in official statistics or reports on drug use, and as a consequence we have chosen to use unpublished raw data. We use the following databases:

1. National data on drug use among Swedish high school students 2007–2012, taken from the annual self-report surveys on drug use conducted by CAN (Centralförbundet för alkohol- och narkotikaupplysning—The Swedish Council for Information on Alcohol and Other Drugs), a non-governmental organization conducting nationwide anonymous surveys (on the government’s behalf) at a representative selection of Swedish schools. Each year some 4,000 pupils in year 9 (aged 15) of compulsory school, and some 4,000 second-year high school students take part. We are using the high school data set only (17 to 18 years of age).

2. Regional data on young drug users in outpatient care 2010–2012, taken from the UngDOK database. UngDOK is a documentation system used in outpatient care of young people (13 to 25 years of age) with varying levels of drug problems. The system is based on structured interviews with the clients. Data are reported from outpatient clinics in Sweden’s three major cities—Stockholm, Gothenburg, and Malmö—as well as a few minor towns [27].

3. National data on adult drug users (18 to 29 years of age) in compulsory treatment 2002–2012, taken from the SiS research registry. SiS (Statens institutionsstyrelse—The National Board of Institutional Care) is a national authority in charge of compulsory treatment and treatment of adolescents with severe drug problems and/or psychosocial problems, and of adults with a life-thretening addiction. SiS is running a large number of institutions all over the country. The authority uses the ADAD (Adolescent Drug Abuse Diagnosis) [28] and DOK [29] documentation systems. All data are gathered in a central research registry.

The analysis of the data, in the form of calculation of frequencies and averages, was conducted in SPSS.

### Qualitative data

Our qualitative data material is made up of semi-structured interviews with professionals who, in their line of work, come into contact with young people with varying degrees of drug use. We have conducted a total of 86 interviews in five Swedish cities; two big cities (Gothenburg and Malmö) and three mid-size towns (Lund, Norrköping and Jönköping). The locations were selected in order to cover a variety of local drug scenes. In bigger cities with many units working with the same populations, we spoke to coordinators and a few randomly selected professionals. The greater part of the interviews took place in the first half of 2012 with some supplementary interviews in the Spring of 2013. The material is summarized in Table 2 below.

**Table 2:** Interviews at differens locations

	**Gothenburg**	**Malmö**	**Lund**	**Norrköping**	**Jönköping**	**Total**
Police (adolescent units and drug squads)	5	3	1	2	2	13
School counselors (high school)	5	4	2	1	4	16
Outreach/social street workers, youth workers, social workers in the police force	2	6	2	2	5	17
Addiction treatment staff (outpatient care, institutional/compulsory care, detox units)	15	10	4	4	1	34
Representatives of associations and NGOs	2	3		1		6
Total	29	26	9	10	12	86

The majority of the interviews took place over the telephone, although a number were also conducted face-to-face or via e-mail. The informants were, among other things, asked about their working methods, the target populations they encounter as well as which narcotic substances or classified prescription drugs are the most common among these target populations. Following on from this, we asked more specific questions about methadone or buprenorphine: whether these substances were used by the adolescents and young adults that the informants come into contact with, whether they could see any change or trend over time, and finally, their own views on diversion and illicit use.

The more extensive interviews were digitally recorded and transcribed verbatim. The analysis was performed as a manual, three-step qualitative textual analysis. First, we made a close reading of the material, and performed a summary coding based on the overarching themes in the interview guide. Then we made a second, more detailed, coding, where we identified various patterns—similarities and differences—in relation to the original. Finally, we went through the material one last time in order to identify suitable illustrative and representative quotes.

### Ethical considerations

The study forms part of a larger research project on diversion and illicit use of methadone and buprenorphine in Sweden, financed by the Swedish Council for Working Life and Social Research. ^b^The project is conducted in accordance with the Swedish Ethical Review Act (SFS 2004:460), and has been reviewed and authorized by the Regional Ethical Review Board at Lund University (dnr 2011/763).

The quantitative data used for this sub-study were de-identified. The CAN drug habit survey was performed anonymously, and as far as the UngDOK and SiS databases are concerned, we have only had access to information about gender, age and drug experiences of the clients.

The interviewees in the qualitative parts of the study have been informed about the study and its aims, as well as their right to end their participation at any time. In the article, their quotes have been anonymized.

## Results

### High school students—few are aware of methadone and buprenorphine, and extremely few have tried the substances

Data from CAN’s high school survey of 2012 show that some 20% of boys and 15% of girls in the second year (17–18 years) stated that they had tried narcotics at some point. The most common substance by far for both sexes was cannabis (in total approx. 16%), followed by benzodiazepines (some 2%) amphetamine, ecstasy and cocaine (approx. 1% each), LSD (0.5%) and heroin (0.3%).

Methadone and buprenorphine are not specified in the CAN surveys, but the surveys do include open-ended questions where the respondents can enter any other narcotic substances they have used, including any prescription drugs they have used in combination with alcohol for intoxication purposes. Between 2007 and 2012, a total of 26,848 high school students answered the questionnaire on drug use. Other narcotic substances have together totaled approx. 1% each year, and each year somewhere between 5% and 8% of the respondents have claimed that they have used some form of prescription drug together with alcohol in order to increase intoxication [30]. During the entire 2007–2012 period, only four high school students stated that they had used buprenorphine. Methadone does not appear even once in these data. As a notable comparison, during the same period 53 students said they had used tramadol (a mild opioid analgesic).

Interviews with counselors at high schools in the five locations investigated indicate similar results. Very few high school students are caught in possession of, or test positive for narcotics. ^c^In a large majority of cases where young people have tested positive for narcotics, or have admitted to drug use, the substance has been cannabis. In recent years a small number of cases of cocaine, amphetamine, and benzodiazepine use have been detected.

None of the counselors interviewed have personally come across any confirmed cases of methadone or buprenorphine use, nor have they heard of any colleagues who have done so. The following quote is representative of our school counselor interviews:

Over the years, cannabis has been the most common [classified] drug by far, it is almost exclusively a case of cannabis. Only a small number test positive for other substances. Having said that, tramadol has increased in recent years—it seems to get more and more common. We have never seen methadone or buprenorphine ^d^in the drugs tests, and I personally have never come across them during all my time as a high school counselor. I doubt that more than a handful of high school students are even aware of these substances. (School Counselor Co-ordinator for Malmö’s municipal high schools)

Tramadol is mentioned in this quote. This is the prescription drug most often mentioned in the interviews. A number of other counselors confirm that tramadol have become more common in recent years, although other classified prescription drugs are mentioned as well, for instance various types of analgesics, sleeping pills, and ADHD medication.

### Young people who use illegal drugs—few have tried methadone or buprenorphine

Outpatient clinics for young drug users is found in Sweden’s three major cities—Stockholm, Gothenburg, and Malmö—and in some smaller towns. The clinics are open for adolescents and young adults through to the age of 25. The target population is broadly defined and encompasses everyone from 13 year-olds, who turn up accompanied by a parent after their first brush with alcohol or other drug experience, to young adults with a more established drug problem. The majority of the visitors, however, are not characterized by long-standing or advanced drug use.

The clinics employ a new, joint documentation system, UngDOK [27,31]. Between 2010 and 2012, a total of 2,003 visitors were registered at the outpatient clinics in the three major cities. Cannabis was the most common narcotic substance used by adolescents at some point (86%), followed by amphetamine (14%), benzodiazepines and cocaine (12% each), ecstasy (7%), methadone/buprenorphine (2.2%), GHB (1.9%), and heroin (1.5%). A substantial proportion of adolescents (19%) furthermore claimed they had used some other drug, not specified on the questionnaires, mainly in the form of various ‘designer drugs’ (new synthetic drugs).

The great majority of adolescents/young adults stated cannabis (64%) or alcohol (26%) as their main drug. Only two people mentioned buprenorphine as their main drug, and one person methadone.

Out of the 45 visitors (2.2%) who had used methadone/buprenorphine, 32 had used buprenorphine only, seven methadone only, while six had tried both methadone and buprenorphine. The average age for methadone initiation was 16.3 years, and 17.1 years for buprenorphine. A small number of adolescents, however, made their debut with one or the other of these substances before they had turned 15. No one mentioned methadone or buprenorphine as the first drug tried, and the majority had used a number of other narcotic substances before trying methadone or buprenorphine. On the other hand, a majority had used methadone or buprenorphine before they tried heroin.

The great majority of those who at some point had used methadone or buprenorphine, reported no use within the last three months. Only three people had used either of the substances extensively (two to three days per week or more) within this period.

A number of representatives for the professions interviewed encounter adolescents and young adults who are using illegal drugs (see Table 1). These professionals meet several thousand adolescents and young adults every year.

On the whole, the interviewees outline similar observations. Cannabis is the dominant narcotic substance among the young people they meet. Few of them have used ‘hard drugs’, such as amphetamine, cocaine, or heroin. That said, various synthetic substances and ‘designer drugs’ are also relatively frequent, and seems to be growing increasingly common according to several informants.

The classified prescription drugs encountered are mainly benzodiazepines, tramadol and codeine. Buprenorphine and methadone, on the other hand, are not described by anyone as common among the young people they come into contact with, nor do they appear to have increased in recent years. Those who have encountered young people who have tried either of these two substances, say they are limited to a few cases each year. Young people who try methadone and buprenorphine have, according to the informants, often tried a number of other drugs previously, and have other drugs as their substance of choice. In the words of a member of the treatment staff at an outpatient clinic in Malmö:

For the adolescents and young adults who come to us, cannabis is the most common problem by a long stretch. Then comes tramadol, followed by benzodiazepines. We very rarely come across young people who have used methadone or buprenorphine. As a result of the debate about diversion lately, we have acquired a test strip for methadone, to be able to screen for it. We have been screening for buprenorphine for a long time. So far we’ve had no positive cases. (Outpatient care unit for young people, Malmö)

In a few cases, professionals have encountered 14- to 16-year olds who have been buprenorphine or methadone positive, and a couple of interviewees describe how young people have developed an addiction to either of these substances. They describe cases where young people have overdosed on methadone or buprenorphine, and one informant told us of one teenager who died following a methadone overdose. Such cases, however, are rare exceptions.

Generally speaking, classified prescription drugs are more prevalent than narcotic substances such as amphetamine, cocaine, and heroin among young drug users. Several informants explain this phenomenon by the fact that many prescription drugs are cheap and easily accessible for adolescents, since they are often to be found at home or in the homes of close friends.

The rarity of buprenorphine and methadone compared to other classified prescription drugs can be explained by the fact that these substances are rarely found in the immediate environment of young people, that most young people are unaware of them, and that access to substitution medication typically require contact with OST-patients or other persons with an opiate or opioid addiction.

Many of the adolescents who come to us, it has be to said, take any drug they can lay their hands on; those who take tramadol and benzodiazepines probably wouldn’t be averse to trying buprenorphine. However, in order to get access to methadone and buprenorphine you need to have to gone further down the addiction road than most of our visitors actually have. Most of our visitors don’t even know what methadone or buprenorphine is when we bring them up in our survey interviews. (Outpatient clinic for young people in Malmö)

To summarize, also when it comes to adolescents and young adults who are drug users, registry data and interviews with professionals paint a consistent picture: methadone and buprenorphine use is highly unusual.

### Adolescents and young adults with a severe drug problem—many have tried methadone and buprenorphine, few use them as their main drug of choice

Information about young people with severe drug problems is to be found in statistics from Statens institutionsstyrelse (SiS, The National Board of Institutional Care), the Swedish government agency that delivers compulsory care for young people with psychosocial problems (the LVU-act) and for adults with a life-threatening addiction (the LVM-act). Since there are no data on the use of methadone and buprenorphine, respectively, for those adolescents who are taken into care under the LVU act, we have relied exclusively on data about young adults who have been taken into care under the LVM act.

Each year, some 1,000 adults are received into care at LVM homes. Typically, the detainees have a history of several years of drug addiction and in a majority of cases detention at an LVM home is the result of immediate action in a life-threatening situation (SiS 2012).

The statistics for the 18 to 29 age bracket for people in LVM care in 2011 (n=290), indicate extensive and advanced drug use: 93% had used cannabis, 86% amphetamine, 73% ecstasy, 66% heroin, and 66% cocaine. Methadone had been used by 54%, and other opiates/opioids by 66%. There is no breakdown of the individual substances included in the latter category, nor any data of how many had used buprenorphine. Of the methadone users, the overwhelming majority (88%) had not undergone OST, a sign that the use in most cases was illicit.

Data from the annual SiS report from the years 2002–2013 show a gradual increase in the proportion of detainees having used methadone, from approx. 30% in 2002 to just over 50% in 2012. Over the last 10 years the average initiation age for methadone was 20–21 years, while the average initiation age for heroin was 18–19 years. Of all narcotic substances, methadone was the one with the highest average initiation age.

Our interviews with professionals who come across young people with severe drug problems include police officers in street dealer units, as well as staff at detoxification units, treatment clinics for adults with an addiction, and institutional and compulsory care homes for young people (LVU homes) and adults (LVM homes).

According to our informants, illicit use of methadone and/or buprenorphine is relatively common in this group. The use of these substances appears to be especially prevalent among adolescents and young adults who are taken into compulsory care. However, methadone and buprenorphine are not described as being among the most commonly used substances by any of the professionals. In this target population of drug users with more severe problems, cannabis, amphetamine, and heroin dominate as the primary drugs of choice. Classified prescription drugs, such as tramadol and various benzodiazepines, also feature more regularly than methadone and buprenorphine.

Those who have used methadone or buprenorphine are mostly above the age of 20, and have a long history of drug use, according to the informants, although they also point to examples of younger people (15 to 17 years) who have used the substances. The great majority, however, have previously used other opiates/opioids, and are not using methadone or buprenorphine as their main drug of choice.

Cannabis is the substance used by most, while amphetamine, cocaine, and heroin are also common. Various classified pills and prescription drugs are very common, mainly benzodiazepines and tramadol, but also buprenorphine. Probably more than half of the young adults we receive here have used methadone or buprenorphine illicitly, most often buprenorphine. Some have used it while in substitution treatment as well, and may have been thrown out, or be on the waiting list to get admitted […] Prior to using methadone or buprenorphine, most have used other opiates, typically heroin or tramadol. (Staff member at compulsory care home for adults with an addiction, Lund)

The professionals mainly point to two categories who use methadone and buprenorphine illicitly. One category consists of people with a heroin addiction, who periodically use methadone or buprenorphine because they are unable to get hold of heroin, because they want to quit heroin, or (as mentioned in the quote above) because they are on the waiting list for, or have been excluded from, OST. The second category consists of people with an extensive polydrug use. This population uses methadone or buprenorphine periodically, usually in conjunction with alcohol or other substances.

Also in the case of adolescents and young adults with the most severe drug problems registry data and interviews indicate a fairly uniform picture. Many members of this population have used methadone and buprenorphine, but very few have them as their main drugs of choice, and, as a rule, these substances enter their drug careers at a late stage.

### Diversion and illicit use—the professionals’ view of the phenomenon

Diversion and illicit use of methadone and buprenorphine is not generally perceived as a widespread or serious problem by the majority of the professionals interviewed. For instance, this is what one of the drug squad officers we interviewed had to say:

I consider the risk, at least considering the situation thus far, of these substances spreading to younger people or non-problem drug users as small […] Clearly there are risks with these substances, that they will reach the wrong people, but I don’t think it’s a big problem. (Officer at the Street Dealer Unit of the Malmö Police)

Several professionals have expressed concerns over what they see as a trend where adolescents increasingly use various classified prescription drugs illicitly. This trend, according to some informants, is associated with an increase in the legal prescription of, and access to, classified prescription drugs, and to a diminished respect for such substances among young people.

Diversion and abuse of buprenorphine and methadone is no more problematic than other classified prescription drugs out there on the streets. The big problem is that it’s easier for a young person today to take a classified pill, and that threshold will just get lower and lower. […] Tramadol, benzodiazepines, and sleeping pills are probably pretty common as gateways to drug abuse for young people today. Many have access to potent prescription drugs at home. There’s not much in the way of restrictions for a number of these substances, not like for buprenorphine or methadone. (RFHL, Riksförbundet för Rättigheter, Frigörelse, Hälsa och Likabehandling—‘The National Association for Rights, Freedom, Health, and Equal Treatment’)

Operations working with drug seizures and/or drugs testing (for instance, the police and the NGO ‘Parents Against Drugs’) have not experienced any great change in the accessibility of methadone and buprenorphine. Only a few informants from treatment clinics describe an increase in adolescent use of these substances in recent years.

The majority of informants from organizations working in the addiction treatment field say that they are aware of diversion from OST, and that there is an established black market for these substances. A general view is that the black market and the illicit use mainly concerns people with a heroin or polydrug addiction. Some voice the opinion that the black market primarily is structured by the demand of people with an opiate/opioid addiction, who either have not been admitted to OST, or have been involuntarily discharged. Illicit use for such ‘self-medication’ purposes is seen by several informants as relatively unproblematic, sometimes even as something positive. Here is an example:

Of those we meet, more or less everyone who has used methadone or buprenorphine has used heroin previously. They are heroin users who, on their own, are trying to cut down on the heroin or prefer buprenorphine or methadone. […] I have met some who save part of their dose and sell it on in order to make some money. Clearly not a good thing, but that’s the way it is. I sympathize with clients who are buying their buprenorphine on the street to feel OK, instead of running around, committing crimes in order to buy heroin. It is, or at least it used to be, difficult to be admitted into substitution treatment in Gothenburg. (Addiction treatment clinic for adults, Gothenburg)

The risk of large-scale diversion of methadone or buprenorphine to young drug users is generally seen as low. Furthermore, the informants do not consider it likely that these substances would work as a gateway to other drugs. Their view is that young people do not have access to methadone or buprenorphine to any particular extent, nor that these substances are in any great demand by them. A staff member at a compulsory care institution explains:

I don’t think that illicit methadone or buprenorphine are creating new drug user populations, no one starts with these substances. They enter their [drug] careers quite late, when the person already has a serious problem with drugs. […] I believe other prescription drugs pose a greater problem, for instance benzodiazepines and tramadol. They are more easily accessible, cheap, and enter the drug user’s life earlier in comparison to buprenorphine, for example. (Staff member at compulsory care home for adults with an addiction)

One risk, however, mentioned by representatives for several professions, is that methadone and buprenorphine may lead to fatal overdoses for people without opiate/opioid tolerance. The substances are generally viewed positively, as long as they are used by the right people for the right purpose, but they are also seen as highly potent and dangerous if they end up in the wrong hands. ‘Illicit use of methadone and buprenorphine may save lives as well as take them,’ as one of the interviewees put it.

## Discussion

Nothing in our results points to methadone or buprenorphine being a major problem among young drug users. The substances appear to be relatively unknown, and very few adolescents and young adults have tried them. Nor is there any evidence to suggest that methadone or buprenorphine are serving as gateway drugs for those who ultimately develop severe drug problems—a claim often made in the Swedish debate.

The ‘gateway model’ is based on well-documented statistical connections where the use of certain common drugs (tobacco, alcohol, cannabis) precedes the use of ‘heavy drugs’, such as amphetamine, cocaine and heroin [32-34]. Earlier and more frequent use constitutes an increased risk for the young user to move on to heavier drugs subsequently. Some researchers and scholars claim that the links of the gateway model are based on causal mechanisms of a biomedical or social nature, although such views are not uncontroversial [35]. Methadone and buprenorphine, however, cannot be considered as gateway drugs in any respect. As substances they enter drug careers at a late stage. In actual fact, methadone or buprenorphine use tends to be a clear indication of a person already having a severe drug problem, rather than a risk factor for establishing such a problem.

There are various possible explanations as to why methadone and buprenorphine are rare among adolescents. Research into diversion and illicit use indicate that the black market for these substances appears to be relatively closed [18,36]. Methadone and buprenorphine are seldom available from street dealers. Instead they are primarily sold by patients in opioid substitution treatment, and the customers mainly consists of persons with an opiate/opioid or polydrug addiction. In comparison to other classified prescription drugs, they are also strictly controlled. Our study indicates that adolescents rarely have access to, or are interested in, this market. A number of the professionals interviewed also voice the opinion that most adolescent are not even aware of these substances.

However, when it comes to adolescents and young adults with severe drug problems, illicit use of methadone and buprenorphine is far more common. In this population there are persons who use these substances as their main drug of choice, but in the majority of cases it is rather a question of polydrug use where methadone and buprenorphine are just two of many of substances.

One population of young drug users stand out in particular—users with a heroin addiction. The great majority of this population have used methadone or buprenorphine. Previous research indicates that heroin addicts mainly use methadone and buprenorphine for self-medication purposes in order to avoid withdrawal symptoms (‘to stay healthy’), to perform self-detoxification or manage opioid substitution on their own [13,14,36]. Our study at least partly confirm these results, but the knowledge of the situation of young people with severe drug problems is still incomplete. Thus more research is needed, for instance qualitative interview studies investigating the users’ motives for using methadone and buprenorphine, and their views on and knowledge of the substances.

As mentioned in the introduction, diversion and illegal use of methadone and buprenorphine is a cornerstone of the criticism often leveled at opioid substitution treatment. In Sweden, the debate has taken the form of an ideological battle between opponents and representatives of this form of treatment [37,38]. However, the voices of professionals who regularly come into contact with young drug users have not been heard.

Diversion and illicit use of methadone and buprenorphine is, in general, not seen as a serious or growing problem by the professionals interviewed. Nonetheless, some treatment staff point out that for a small group of young people, the substances may form part of a dangerous polydrug use, and that they can cause fatalities among young people without sufficient knowledge of or respect for these substances. Accordingly, from a harm-reduction perspective, it is reasonable to concentrate on interventions based on informing young drug users about the risks of these substances. Important target groups for such interventions are adolescents and young adults in institutional and compulsory care, as well as young drug users in outpatient treatment.

Measures to minimize diversion of methadone and buprenorphine, and to target the illicit use of these substances is an important issue. The design of such measures, however, present difficult trade-offs, since the advantages of offering easily accessible, effective, and user-customized treatment for a large group of individuals must be weighed against the negative effects of diversion. The fact that the customers on the illicit market mainly consist of opiate or opioid addicted individuals, many of whom are using the substances for self-medication purposes, needs to be taken into account in those trade-offs.

Our results indicate that other classified prescription drugs are used by adolescents to a far greater extent than OST medication. Benzodiazepines and tramadol are extensively prescribed to a broad user population, and are not as strictly regulated as methadone and buprenorphine. Moreover, these prescription drugs enter the drug career of many young users early, and appear to be relatively easily accessible. Research focusing on the illicit market of such substances is needed, as well as measures preventing illicit use.

## Conclusions

The illicit use of methadone and buprenorphine among young drug users is not a widespread problem in Sweden. Among adolescents and young adults with severe drug problems, however, methadone and buprenorphine is more common, and may cause dangerous health risks. Harm-reduction measures, such as information about overdose risks, should therefore target those groups. Prescription drugs such as benzodiazepines and tramadol, which are less controlled and more widely used among young people, is of greater concern. Research focusing on the illicit market for these substances, their possible role in drug careers and their potential negative consequences for users is needed.

## Endnotes

^a^After many years with low treatment accessibility, OST has increased substantially in Sweden during the 2000s. Today, there are over 5,000 OST-patients across the country, a coverage rate of 50-75% (current estimates of the number of people with an opiate/opioid addiction are very uncertain). About half of the patients are prescribed methadone, and the other half buprenorphine or buprenorphine/naloxone. The main inclusion criteria for OST are (1) age > 20 years and (2) a documented opiate (heroin or morphine) addiction for at least one year. According to government regulations, individuals with an opioid addiction are not allowed to be admitted to OST. Swedish OST is strictly controlled. During the first six months, all patients are required to attend the clinic daily for supervised medication. After that, control is gradually reduced for compliant patients. Urine tests are taken regularly. Patients who do not comply with treatment regulations run the risk of being discharged, resulting in a three-month “lockout” period.

^b^The project includes an extensive quantitative interview study with patients undergoing OST and injecting opiate/opioid users, as well as a number of qualitative in-depth studies and registry studies. The main themes explored are (1) the extent of, and motives for diversion from OST, (2) the patients’ views on various aspects of their treatment, (3) the experiences of illicit use of methadone and buprenorphine among various groups of drug users, (4) methodological studies focusing on the opportunities and risks of user involvement in research, and (5) treatment staff views on, and management of, diversion.

^c^In Swedish schools, drugs tests are performed in the event of suspected drug use. A few schools also conduct random drugs tests even without reasonable suspicion.

^d^Most of our informants use the medical product name, Subutex, but in the quotes we have altered this to the substance name throughout.

## Competing interests

The authors declare that they have no competing interests.

## Authors’ contributions

BJ planned the original project. BJ and TR designed the study and did the interviews. TR conducted most of the analysis and wrote the first draft. Final revisions were made jointly by BJ and TR. Both authors read and approved the final manuscript.
